# Quadrant and Dermatomal Analysis of Sensorial Block in Ultrasound-Guided Erector Spinae Plane Block

**DOI:** 10.5152/eurasianjmed.2022.21151

**Published:** 2022-06-01

**Authors:** Onur Selvi, Serkan Tulgar, Talat Ercan Serifsoy, Robert Lance, David Terence Thomas, Yavuz Gürkan

**Affiliations:** 1 Department of Anaesthesia, Anesthesiology and Reanimation, Addenbrooke’s Hospital, Cambridge, Cambridgeshire, UK; 2 Samsun University Faculty of Medicine, Samsun Training and Research Hospital, Samsun, Turkey; 3 Department of Medical Education, Maltepe University Faculty of Medicine, İstanbul, Turkey; 4 Department of Anesthesiology and Reanimation, Koç University, İstanbul, Turkey

**Keywords:** Anesthesiology, perioperative and adult anesthesiology, postoperative period, regional anesthesia, erector spinae block

## Abstract

**Objective:** As a novel procedure now gaining popularity, erector spinae plane block has been the subject of many studies. However, dermatomal coverage of the sensory block caused by erector spinae plane block has been rarely studied. The goal of this study is to evaluate the sensory block resulting from erector spinae plane block applied at the T9 vertebral level.

**Materials and Methods:** This observational, prospective, blinded study was conducted on 50 adult patients undergoing laparoscopic abdominal surgery. All patients underwent bilateral erector spinae plane block at the T9 level after completion of the surgery while under general anesthesia. In order to further evaluate the sensory blockade, we divided the hemiabdomen–hemithorax region into 4 quadrants: dorsal-medial, dorsal-laterel, ventral-lateral, and ventral-medial. The sensorial evaluation was performed using the pinprick test, 2 hours following the application of erector spinae plane block.

**Results:** A total of 28 female and 22 male patients were examined in this study. Complete failure of the block was recorded in 7 patients, with no thoracic/lumbar segmental or quadrant involvement. Successful sensory block was achieved in 67% of the dorsolateral quadrants, 58% of the dorsomedial quadrants, 69% of the ventrolateral quadrants, and 55% of the ventromedial quadrants.

**Conclusion:** Cutaneous sensory block of erector spinae plane block at T9 vertebral level revealed variable results and low failure rates. Administration of erector spinae plane block for postoperative analgesia in thoracoabdominal surgeries requires further randomized controlled trials to confirm its effectiveness and convenience.

**Main Points**

- Cutaneous sensory block of erector spinae plane block (ESPB) exhibits variable results following ESPB applications,
- Erector spinae plane block should be used as a complementary treatment in addition to the multimodal analgesia.
- Application site of the ESPB should be considered carefully regarding desired spread for sensory block following surgical intervention.

## Introduction

The erector spinae plane block (ESPB) was first defined by Forero et al^1^ for the treatment of thoracic neuropathic pain, but the anatomic, radiological, and clinical data obtained in these studies led to the use of this technique in different areas. Erector spinae plane block has become an accepted tool in chronic pain syndromes, acute pain, and postoperative pain.^2-4^

While there are several case reports of ESPB, there is a limited number of randomized controlled studies on ESPB.^3,5^ In the literature, dermatomal coverage of sensory block caused by ESPB has been reported only rarely.^2,6,7^ Cadaveric and imaging studies performed to determine the anatomic spread of local anesthetic (LA) in ESPB have demonstrated differing results leading to continued uncertainty regarding the mechanism of this block.^8-12^ While quadrant evaluation has been reported in correspondences, there are no case series or clinical studies evaluating the dermatomal and quadrant sensory block of ESPB.^13,14^ The aim of this study was to evaluate the dermatomal and quadrant sensorial blockage generated by ESPB when performed from T9 vertebral level after laparoscopic abdominal surgeries.

## Materials and Methods

This observational, prospective, blinded clinical study was performed at Maltepe University Hospital between November 15, 2018, and October 3, 2019, in accordance with the Declaration of Helsinki principles. The first patient was recruited on November 15, 2018, and the first patient was enrolled on November 19, 2018. The study was approved by the local ethics committee (Maltepe University Clinical Research Ethical Board Approval No: 2018-900-56) and the study was registered with clinicaltrials.gov (NCT03744520). Strengthening the Reporting of Observational Studies in Epidemiology (STROBE) guidelines were followed. All patients gave written informed consent for participation and the use of their data in scientific research.

Patients scheduled to undergo laparoscopic surgery with bilateral ESPB from the T9 vertebral level were recruited for the study. Patients <18 years old, those with a body mass index >35 or <22, those with psychiatric or neurologic disease-causing communication problems, those patients using any medication that would affect the perception of pain, and those using steroids or any additional analgesics such as gabapentinoids or antipsychotic medications preoperatively that were not part of the protocol were excluded from the study. In addition, patients with scar tissues within the sensory evaluation area or having a history of thoracic and spinal surgeries were excluded from the study. Fifty consecutive patients scheduled to undergo bilateral ESPB were to be included, with the aim of evaluating 100 blocks. The primary outcome of this study was to evaluate the sensory block of ESPB in the thoracoabdominal area. Since there are no similar studies in the literature and our results are of descriptive nature, a formal sample size calculation was not performed in this observational study. Statistical analyses were performed by using Number Cruncher Statistical System 2007 and Power Analysis and Sample Size 2008 Statistical Software (Utah, USA).

### General Anesthesia and Postoperative Analgesia

Anesthesia management was identical in all patients. Patients received no premedication. Following standard monitoring, intravenous (i.v.) fentanyl 1.5 μg/kg, propofol 2-3 mg/kg, and rocuronium 0.6 mg/kg were used for induction. Maintenance was achieved using 0.6-1 Minimum alveolar concentration (MAC) sevoflurane. Our standard perioperative analgesia protocol for abdominal surgeries under general anesthesia includes morphine 0.05 mg/kg (maximum 4mg), paracetamol 1 g, and tenoxicam 20 mg i.v. At the end of the operation, the neuromuscular blockade was reversed with 0.04 mg/kg of neostigmine and 0.02 mg/kg of i.v. atropine. Patients were extubated and transferred to the recovery room (RR) when adequate muscle strength had returned. No LA was used by the surgical team.

The same postoperative analgesia protocol was applied to all patients. Following transfer to the recovery area, patient-controlled analgesia (PCA) was commenced. Intravenous PCA included 3 mg/mL of tramadol with no basal infusion, 10 mg bolus, and a 20-minute lockout time. If the numeric rating scale (NRS)  was ≥4 in RR, then fentanyl 25 μg i.v. bolus was applied and repeated every 20 minutes until NRS <4. Therefore, no patient was left without analgesia until i.v. PCA and ESPB provided adequate pain relief.

In the ward, in addition to PCA, paracetamol 1 g i.v. was scheduled every 8 hours routinely, with the first dose 8 hours after its intraoperative administration. If patients’ NRS was ≥4, intramuscular diclofenac NaCl 75 mg was used as rescue analgesia. However, evaluation of NRS scores or pain status of the patient is not in the scope of this study and was not evaluated.

### Erector Spinae Plane Block Application

All patients underwent bilateral ESPB under sterile conditions after completion of the surgery, while under general anesthesia. Patients were placed in the lateral position, and a linear ultrasound transducer was placed 2.5-3 cm lateral to the T9 spinous process in a parasagittal orientation. A 22G 5-8 cm needle (BRAUN Stimuplex A®, Germany) was inserted using an out-of-plane approach on the outer surface of the ultrasound probe. The needle was advanced toward the tip of the transverse process and the location of the needle tip was confirmed with hydrodissection. About 30 mL of bupivacaine 0.25% was injected in between the transverse process and erector spinae muscle. The same procedure was repeated for the opposite side. Erector spinae plane block was performed by the same experienced anesthesiologist (ST) in all patients.

### Sensorial Evaluation

The sensorial evaluation was performed using the pinprick test 2 hours after ESPB was performed. This timeframe ensured that LA had bound to tissue and that patients were fully awake for evaluation. Patients’ level of sedation was controlled using Ramsay Sedation Score, and evaluation of sensorial block was postponed until adequate communication was established. Starting from higher-level thoracal dermatomes, the assessor ensured that the patient reports the sensation of pain rather than that of pressure. The same anesthesiologist (OS) performed all sensorial evaluations of the blocks using a standard chart in the postoperative care unit or the ward. Sensorial analysis of regional fascial blocks is routinely performed at our institute and we have been documenting the quadrant analyses of all interfascial plane blocks. The assessor, therefore, was not aware of the block applied and used a standard evaluation chart to examine dermatomes. This assessor evaluated sensorial innervation in 4 separate quadrants: ventromedial, ventrolateral, dorsomedial, and dorsolateral. This grouping was based on the assumed formation of the posterior and the anterior nerve roots and their branches although this varies individually. Complete failure of the block was defined as no thoracic/lumbar segmental or quadrant involvement on sensorial evaluation. The assessor was routinely blinded to the types and levels of all truncal and regional fascial blocks.

## Results

**Figure 1.**
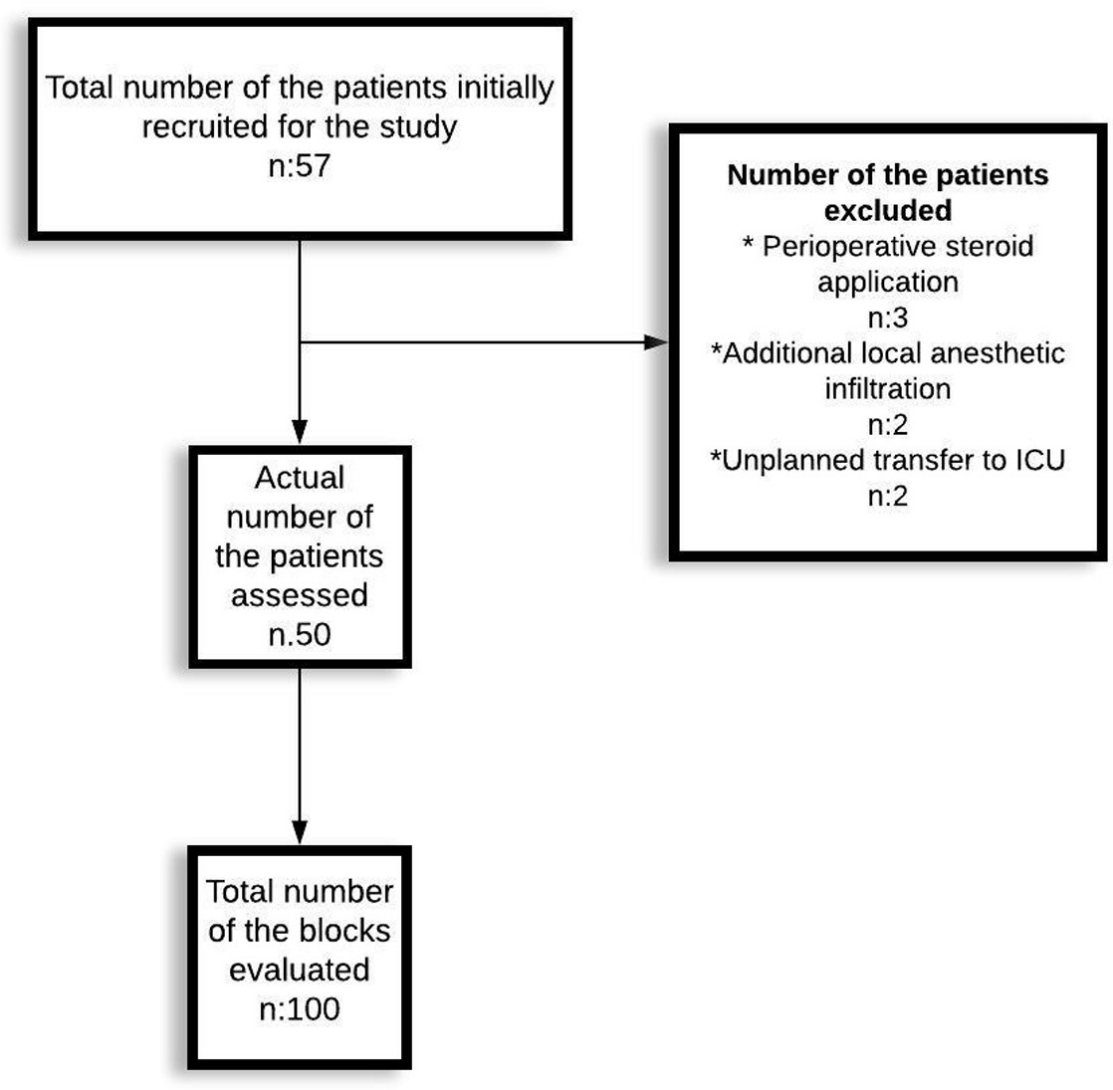
Strobe flow chart. ESPB, erector spinae plane block.

Fifty-seven patients were evaluated for inclusion in the study. Of these, 3 were excluded for perioperative steroid use, 2 for local anesthesia infiltration of the surgical wound, and 2 for being admitted to the intensive care unit due to postoperative respiratory problems. A total of 50 patients were included in the study. With 50 right-sided blocks and 50 left-sided blocks, we included a total of 100 blocks. Details are shown in the STROBE diagram (Figure 1).

Demographic data are shown in Table 1. Surgical procedures included laparoscopic cholecystectomy, Nissen fundoplication, gynecological procedures, umbilical or incisional hernia repair, nephrectomy, and gastric tumor surgery.

**Figure 2.**
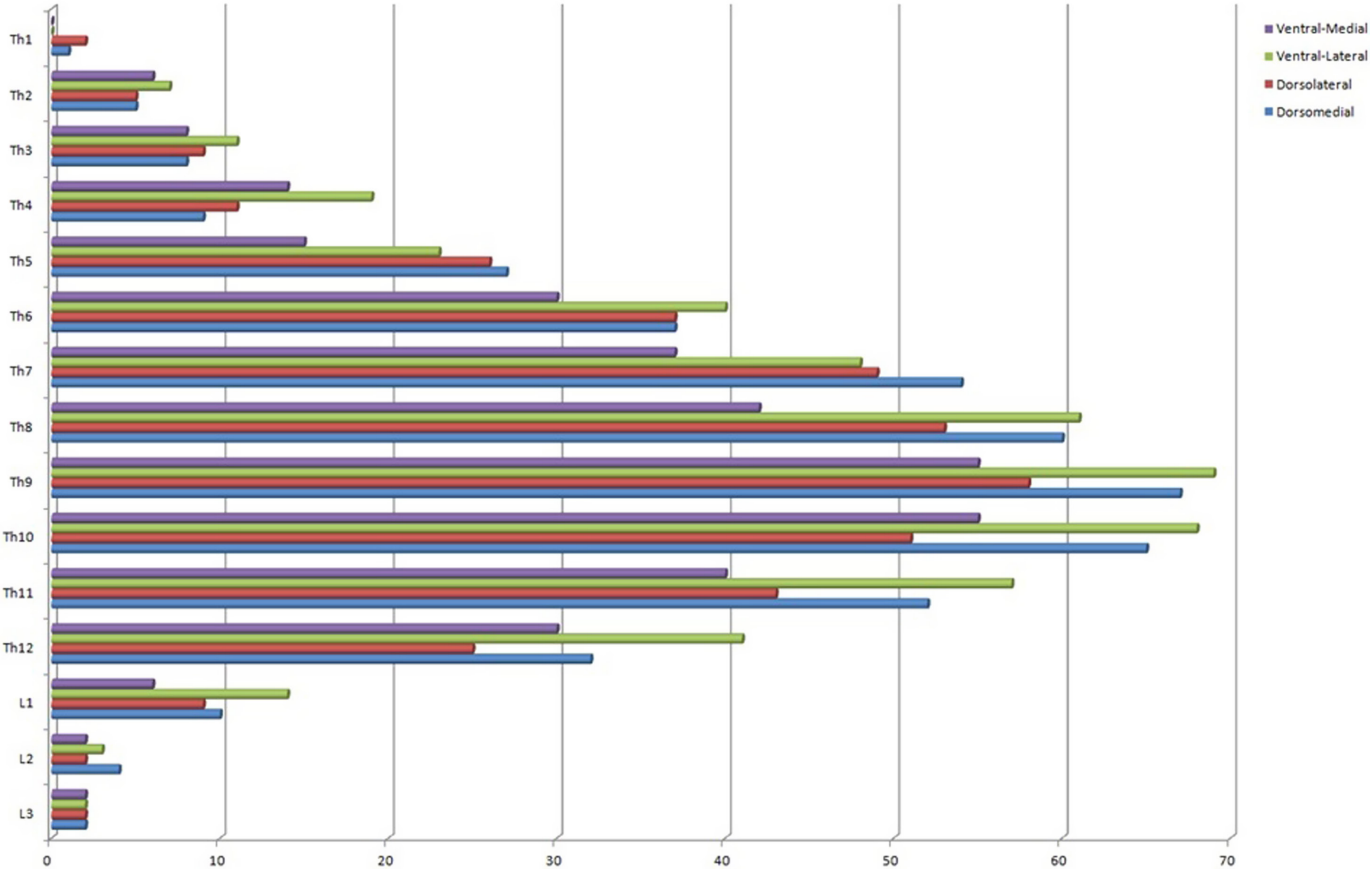
Quadrant involvement of sensorial block distribution of the ESPB at Th9 level. The horizontal axis shows the percentage of the number of the patients who had dermatomal block. The vertical axis shows the thoracal and lumbar dermatomes analyzed with pinprick tests. ESPB, erector spinae plane block.

Complete failure of the block was observed in 7 blocks out of 100. When sensorial blocks were evaluated per quadrant, successful involvement of the specific quadrant was as follows: 67% for dorsomedial, 58% for dorsolateral, 69% for ventrolateral, and 55% for ventromedial quadrants for blocks applied at Th9. Below L1 and above Th5, the sensory block involvement rate was 30%, with the rate decreasing as the distance from the application point increased (Table 2, Figure 2).

No patient had symmetrical dermatomal and quadrant involvement. Success rates for sensorial blocks are demonstrated in Figure 2.

**Figure 3.**
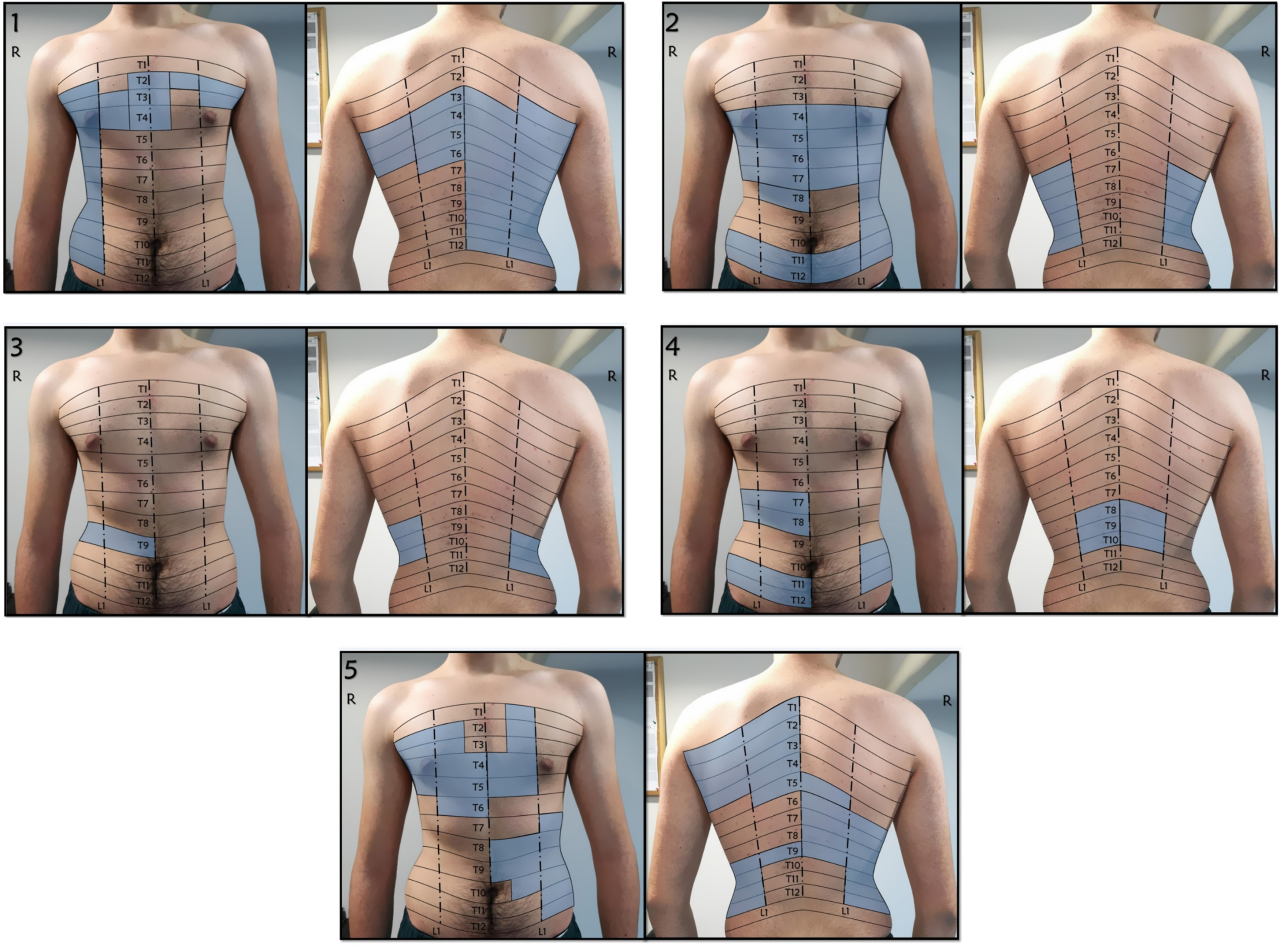
Photo 1-2-3-4-5. Examples of unexpected or patchy involvement following ESPB at T9 level. ESPB, erector spinae plane block.

Unexpected and skipped involvements are shown in Figure 3. In some applications, the sensorial block was observed to skip 1-2 levels with a block observed above and below these levels. In some cases, the sensory block was neither observed in the dermatome of the vertebrae to which the block was applied nor in the adjacent dermatomes, but the effective sensory block was noted in more distant dermatomes. Some examples of unexpected or patchy involvements are shown in Photos 1 through 5.

## Discussion

The results of our study showed differences in both quadrant and dermatomal involvement in the sensory block created by bilateral ESPB applied at the Th9 vertebral level. When applied at this level, the success of ventromedial and dorsolateral quadrant involvement was low, while a higher success rate was observed in the ventrolateral and dorsomedial quadrants. Complete failure was observed in 7 out of 100 ESPBs (%7) and involvement in any dermatome or quadrant was not higher than 70%.

Unusual, unpredictable, and patchy sensory blocks may be due to the unpredictable spread of LA. We do not know whether the LA used in our patients has spread through the vertical plane to the paravertebral–epidural area or through the interfacial plane to the lateral cutaneous branches. Moreover, even if LA passes into the paravertebral–epidural area or reaches the intercostal nerve, it is not guaranteed to surround the nerve or nerve root.

There are other mechanisms that may explain the unpredictable sensory blocks we documented..^15-16^ Adhesions in the interfascial area may lead to blocking failure at the applied dermatome. However, intrafacial adhesions may also cause increased spread to more distant, unexpected sites.^17^ Even if ESPB leads to a sensorial block of the thoracic/lumbar nerve, it may not lead to the same success in the ventral area as there are intersections of intercostal nerves and cutaneous nerve branches distally.^18^ The unexpected and patchy blocks might be a proof of the unforeseen nature of ESPB by showing the abrupt sensorial block dissemination following a block application. Further anatomical studies may explore this unforeseen.

The effect of ESPB on both visceral and somatic pain in abdominal surgeries has been reported.^19,20^ Of note, only a few unsuccessful block applications were published.^2,21,22^ However, the definition of a failed or unsuccessful block needs more attention. The area of sensory coverage documented in our study may represent the dermatomes for which ESPB can provide analgesia. However, in terms of success or failure, the evaluation of analgesic quality that patients perceive might be more fundamental and different. Unfortunately, we neither measured NRS values nor the analgesia needs of the patients. Questions regarding the depth and quality of analgesia resulting from ESPB might be answered with further studies that include other parameters such as patients’ satisfaction and overall narcotic use, for example.

How should block failure/incomplete block be defined in interfascial plane blocks? Should an ESPB that misses the mid-abdominal region be considered as a failed block when it provides postoperative analgesia for vertebral surgery? The same distribution would be considered as inadequate in ventral hernia surgery. Therefore, evaluating ESPB with dermatomal distribution alone may not be appropriate. It is difficult to describe the success or failure of a block without reference to a particular surgical procedure.^22^ The definition of an “inadequate spread for surgical procedure” may be used for ESPB in patients who experience pain in a small proportion of the surgical incision or in patients suffering from reflected pain, even if they do not complain of severe pain.

It may be useful to remind ourselves about the clinical use of peripheral nerve blocks and interfascial blocks. Firstly, interfascial plane blocks should not be considered as peripheral nerve blocks. Although the effectiveness of interfacial plane blocks varies according to the type of surgery, these blocks reduce opioid use and the need for additional analgesics when used as part of multimodal analgesia. This clinical success is an important motivation for continuing these procedures. It is important to bear in mind that an increase in the success of an interfascial block can be achieved by bilevel injections or different modifications,^23-25^ possibly producing superimposed areas of effect.

It should not be forgotten that even in the vertebral dermatome where ESPB is applied, the sensory block success rate of all 4 quadrants was found to be below 60%. We know that the success rate in ventrolateral and dorsomedial is higher in ESPB application from Th9 and that there may be differences between the planned spread of LA and the actual spread. We suggest that ESPB is a good option as a recovery analgesia technique and also can be an effective and safe component of multimodal analgesia rather than being a sole provider of postoperative analgesia.

## Limitations

Primarily, ESPB practitioners use different modifications, and the results of the sensory analysis for each modification can be expected to be different.^26^ In our study, no randomization was performed according to the demographic characteristics of the patients. This condition excludes the examination of variables, which may affect drug distribution, such as surgical procedure, obesity, previous surgery, gender, and muscle structure.^2,7,27^ Therefore, in terms of homogenization, it would be appropriate to perform the block in a uniform patient group.

We have been practicing ESPB using the out-of-plane technique even though it was described as an in-plane technique. The out-of-plane technique has been reported by numerous authors in recent years. As both techniques target the transverse process with the tip of the needle, we are comfortable with the out-of-plane approach. While we do not have any scientific data, it should be kept in mind that the preferred technique may have an effect on the sensorial involvement.

Considering that a unilateral block could pass to the opposite side, the sensorial analysis of unilateral blocks could also give important data.^28^ However, a bilateral interfascial plane block is required in abdominal surgery and therefore unilateral block was not considered in our patients.

Another issue to bear in mind might also explain variable dermatomal testing. The possible unbalanced distribution of LA due to gravity between the upper and lower regions of the ESPB in the lateral position was not investigated. Naturally, different distribution patterns can be expected to occur with applications of LA in prone, sitting, or other positions. However, this may be the subject of another study. The sensorial analysis would be more appropriate in healthy volunteers who did not receive opioids or non-steroid analgesics. More than 1 sensory evaluation at specific intervals would reveal the duration of the sensory block and provide information about the regression of the block. Another limitation is that all ESPBs are applied at the T9 level. Erector spinae plane block may show different characteristics and dermatomal spread at different levels. Differences in anatomical structures both in the upper thoracic, lower thoracic, and lumbar regions may lead to the varying passage of the LAs into the ventral or epidural areas. Therefore, the relationship between block application levels and dermatomal spread requires further evaluation.

When applied at the T9 level, a higher success rate was observed in the ventrolateral and dorsomedial quadrants. However, the cutaneous sensory block is found to be variable following ESPB applications. This finding confirms earlier anatomic/radiological studies. The role of ESPB for postoperative analgesia in thoracoabdominal surgeries should be reviewed with extensive randomized controlled trials and sensory evaluations.

## Figures and Tables

**Table 1.** Patients Descriptive Data

	Mean ± SD	**Minimum-Maximum**
**Age (years)**	42.2 ± 8.62	20-82
**Height (cm)**	166.25 ± 15.16	150-183
**Weight (kg)**	69.94 ± 0.64	60-93
**ASA I/II/III**	26/20/4	
**Gender F/M**	28/22	
**Type of surgery (n)**	Laparoscopic cholecystectomy 18 Laparoscopic Nissen fundoplication 19 Laparoscopic gynecological surgery 4 Laparoscopic umbilical/incisional hernia repair 5 Laparoscopic nephrectomy 2 Laparoscopic gastric tumor surgery 2	

SD, standard deviation.

**Table 2.** Sensorial Block Involvement of Dermatomes

	**Dorsomedial (%)**	**Dorsolateral (%)**	**Ventral-Lateral (%)**	**Ventral-Medial (%)**
**Th1**	1	2	0	0
**Th2**	5	5	7	6
**Th3**	8	9	11	8
**Th4**	9	11	19	14
**Th5**	27	26	23	15
**Th6**	37	37	40	30
**Th7**	54	49	48	37
**Th8**	60	53	61	42
**Th9**	67	58	69	55
**Th10**	65	51	68	55
**Th11**	52	43	57	40
**Th12**	32	25	41	30
**L1**	10	9	14	6
**L2**	4	2	3	2
**L3**	2	2	2	2
**Failed Block**	**23**	**32**	**13**	**31**
